# An Investigation of Bilateral Symmetry in Softball Pitchers According to Body Composition

**DOI:** 10.3389/fspor.2022.868518

**Published:** 2022-07-14

**Authors:** Kenzie B. Friesen, Angelica E. Lang, Karen E. Chad, Gretchen D. Oliver

**Affiliations:** ^1^Canadian Centre for Health and Safety in Agriculture, College of Medicine, University of Saskatchewan, Saskatoon, SK, Canada; ^2^College of Kinesiology, University of Saskatchewan, Saskatoon, SK, Canada; ^3^Sports Medicine and Movement Laboratory, School of Kinesiology, Auburn University, Auburn, AL, United States

**Keywords:** asymmetry, softball, pitch, body fat percentage, range of motion, isometric strength

## Abstract

**Introduction:**

High body fat percentage (bf%) is considered a potential injury risk factor for softball pitchers amidst the already high rates of pitching-related injury. Similarly, research points out that large bilateral asymmetries are another risk factor for softball pitchers. As softball pitching is a highly asymmetric sport and the repetitive nature of the windmill pitch places high stress on the body while pitchers are in unbalanced and asymmetric positions, research examining body composition and asymmetry is necessary.

**Purpose:**

The purpose of this study was to compare functional characteristics of softball pitchers with a healthy and a high bf%. Bilateral symmetry was assessed for pitchers' hip and shoulder isometric strength (ISO) and range of motion (ROM) between the following two groups of softball pitchers: (1) those with a high bf% (≥32%) and (2) those with a healthy bf% (<32%).

**Methods:**

A total of 41 high school female softball pitchers from the southern United States agreed to participate (1.69 ± 0.07 m, 76.14 ± 17.08 kg, 15.1 ± 1.1 years). Pitchers completed a dual-energy X-ray absorptiometry (DEXA) scan and were grouped into one of the following two categories based on their bf%: healthy (<32 bf%) and high (≥32 bf%). Bilateral symmetry was assessed for pitchers' hip and shoulder ISO and ROM using a handheld dynamometer and inclinometer, respectively. Bilateral arm bone and lean mass was also measured *via* the DEXA.

**Results:**

Mixed analyses of variance revealed a significant interaction between bf% groups and side dominance for internal rotation shoulder ROM, F_(1, 39)_ = 14.383, *p* < 0.001, η^2^_*p*_ = 0.269. Main effects for side dominance were also observed for shoulder external rotation ISO, F_(1, 39)_ = 8.133, *p* = 0.007, η^2^_*p*_ = 0.173, hip internal rotation ISO, F_(1, 39)_ = 4.635, *p* = 0.038, η^2^_*p*_ = 0.106, arm bone mass, F_(1, 39)_ = 38.620, *p* < 0.001, η^2^_*p*_ = 0.498, and arm lean mass, F_(1, 39)_ = 101.869, *p* < 0.001, η^2^_*p*_ = 0.723.

**Conclusion:**

Asymmetries and slight differences in functional characteristics exist between bf% groups. Altered functional characteristics may influence pitchers' windmill pitch movement and should be acknowledged by support staff to improve softball pitchers' health and longevity.

**Implications:**

Insight into asymmetries can help researchers and clinicians understand the implication of excess body fat and further theorize mechanisms of injury among this athlete population.

## Introduction

Softball pitching is a highly asymmetric sport, with dominant and nondominant limbs performing drastically different motions (Fuchs et al., 2019). The regular asymmetry and vast amount of repetition present in softball pitching (Corben et al., 2015; Skillington et al., 2017) can lead to adaptations in physical and functional characteristics, as well as altered bilateral movement patterns (Friesen et al., 2019; Hellem et al., 2019). Research highlights the high demand of baseball and softball pitching and reports physical adaptations [e.g., glenohumeral internal rotation deficit (GIRD)] that ensue, due to the repetitious, irregular motions, and high amounts of force repeatedly stressing the body while in specific positions (Kettunen et al., 2000; Robb et al., 2010; Shanley et al., 2011; Li et al., 2015; Zeppieri Jr et al., 2015; Picha et al., 2016; Greenberg et al., 2017; Camp et al., 2018). While research consistently points out that the GIRD present among baseball throwers as a result of frequent positioning of the throwing shoulder into maximal external rotation (ER) and the high-velocity internal rotation (IR) of the shoulder joint (Garrison et al., 2012; Chou et al., 2018), softball pitchers experience similar motions whereby they would experience similar soft tissue and functional adaptation. During the acceleration phase of the softball pitch, pitchers experience high shoulder IR velocity (4,500 ± 1,200°/s), similar to their baseball counterparts (6,703 ± 5,770°/s) (Barrentine et al., 1998). Considering the specific nature of the underhand windmill pitch, understandably there will be adaptations that may vary accordingly. Research dedicated to specifically softball shows that pitchers exhibit greater adaptation in their stride leg than push leg due to the repetitive overloading during each aggressive landing during foot contact of the pitch (Fuchs et al., 2019). Evidence of bilateral differences among pitchers, in conjunction with the various roles of bilateral limbs, highlights the physical adaptations that ensue as a result of repetition.

Altered functional characteristics, such as strength and range of motion (ROM) of major joints, due to high repetition are also widely reported in throwing literature. While altered functional characteristics can bring about necessary adaptations to benefit the athlete, research also shows that some adaptations that evoke drastic asymmetry, such as large bilateral deficits in shoulder internal ROM, may lead to an increased risk of injury (Scher et al., 2010; Shanley et al., 2011; Saito et al., 2014; Tainaka et al., 2014; Bedi et al., 2016; VandenBerg et al., 2017). Therefore, while adaptations might be necessary from a performance perspective, large-scale adaptations noticeable *via* bilateral comparison might warrant caution for athlete safety. Understanding what might lead to greater bilateral deficits is important for ensuring player health and development.

A particular body trait that might pose further threat to asymmetry is body composition. Body fat percentage (bf%) and body mass index have been linked with altered hip and shoulder ROM (Kettunen et al., 2000; Friesen et al., 2020a). Similarly, recent research also suggests that bf% alters softball pitching kinematics and kinetics (Friesen et al., 2020b, 2022; Friesen and Oliver, 2021; Friesen K. et al., 2021). Given the higher rate of injury among those pitchers with greater mass (Oliver et al., 2019a), excess body fat among softball pitchers is a concern. Coincidentally, collegiate softball pitchers display the greatest amounts of bf% among their teammates and reports also show that, on average, they obtain more body fat throughout a competitive season (Czeck et al., 2019; Peart et al., 2019). Pitchers who possess more body fat tissue exhibit higher forces at injury-susceptible joints (Friesen, 2020; Friesen and Oliver, 2021), namely, the shoulder, and therefore, we would expect that there may be more significant joint adaptations for those pitchers with greater mass.

Recent reports show that those pitchers who are injured most often are typically heavier, taller, and have a higher body mass index or bf% (Greenberg et al., 2017; Oliver et al., 2018; Friesen K. B. et al., 2021). Therefore, amidst the already high rates of softball pitching-related injury (Oliver et al., 2019c; Valier et al., 2020), examination into the functional asymmetries of those with various bf% is necessary, especially given that research suggests dramatic bilateral asymmetries can predispose an athlete to greater risk of injury (Shanley et al., 2011). Therefore, the purpose of this study was to compare functional characteristics of softball pitchers with a healthy bf% and a high bf%. Bilateral symmetry was assessed for pitchers' hip and shoulder isometric strength (ISO) and ROM. Furthermore, side-to-side bone mass and lean mass symmetry was assessed for the arms between the two groups of pitchers. It is hypothesized that pitchers within the high bf% group may accrue greater asymmetries in joint ROM and ISO. It was also hypothesized that pitcher groups would display different mean values of bone and lean tissue in their arms.

## Materials and Methods

### Participants

A total of 41 high school female softball pitchers from the southern United States agreed to participate in the study (1.69 ± 0.07 m, 76.14 ± 17.08 kg, 15.1 ± 1.1 years, *n* = 41, *n* = 35 right-hand dominant). Pitchers were grouped into one of two categories based on their total bf%. The cutoff value determining pitchers with high fat% from pitchers with healthy fat% was set at 32% body fat according to the American College of Sports Medicine criterion-based reports, which define 20–32% body fat as being satisfactory for health in women (ACSM, 2013). Therefore, pitchers were grouped in the healthy bf% group if their bf% was <32% and grouped into the high bf% group if their bf% was ≥ 32%. There were 18 pitchers grouped into the healthy bf% group (1.70 ± 0.07 m, 64.54 ± 9.11 kg, 16 ± 2 years) and 27 pitchers grouped into the high bf% group (1.70 ± 0.07 m, 84.04 ± 15.78 kg, 15 ± 2 years).

Prior to participation, all participants were explained the study protocol and informed consent was signed by the participants' parent/guardian while the participant signed assent documentation. To be eligible for participation, pitchers needed to be injury- and surgery-free for the past 6 months and on a current softball roster. They also needed to have pitched in a game within the past 6 months and have reported to the laboratory fully rested for the previous 24 h.

### Procedures

**Figure 1:**
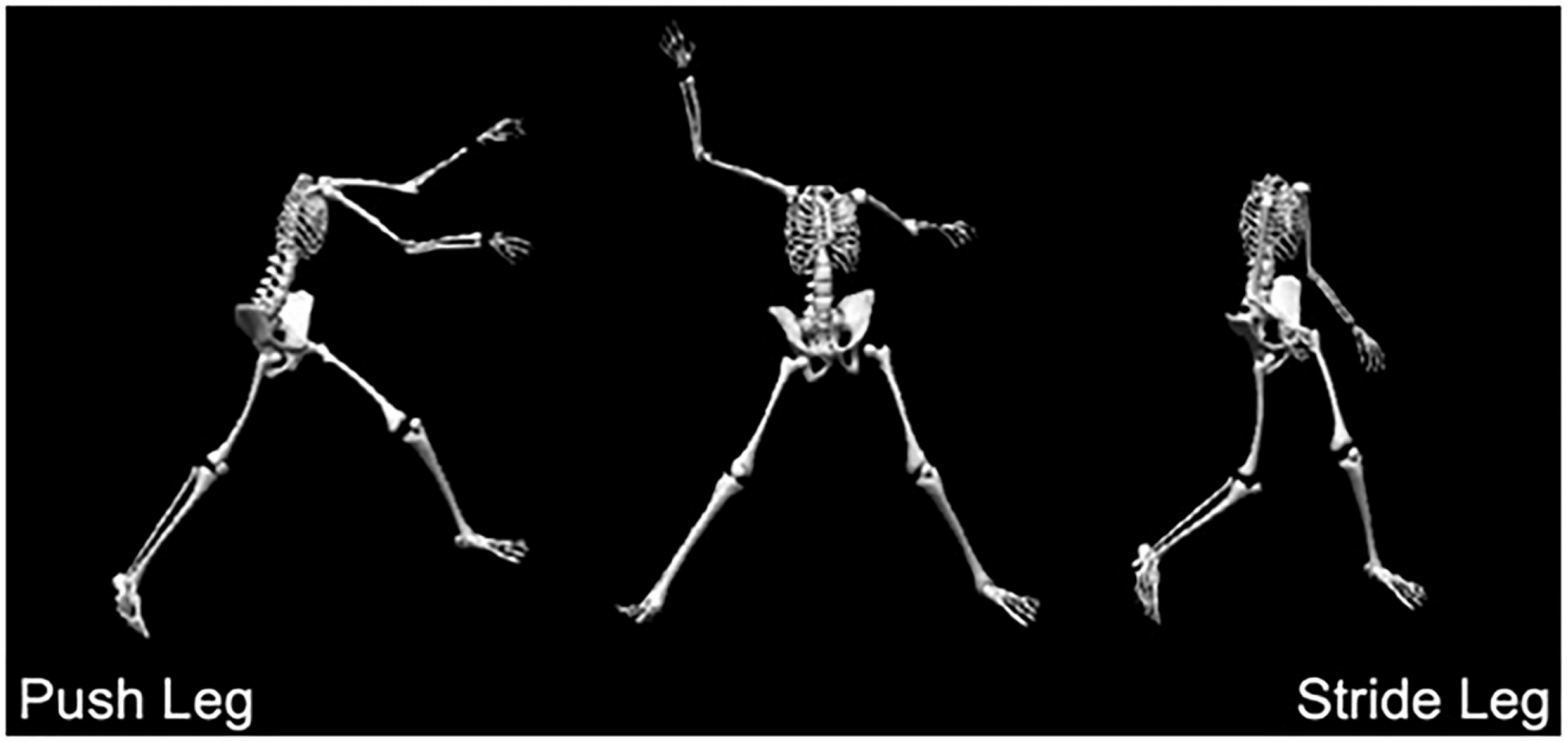
Push and stride leg illustration during the windmill pitch.

All protocols were approved by the Institutional Review Board. Pitchers first completed a dual-energy X-ray absorptiometry (DEXA) whole-body scan, which collected whole-body and segmental composition measurements, including fat tissue, lean tissue, and bone mineral content (GE Healthcare, Madison, WI, USA). The standard error of estimate for the DEXA is ±1.8%. Following the dual-energy X-ray measurement, pitchers' bilateral hip and shoulder ISO and ROM were assessed in both the IR and ER directions. Regarding the hips, the push hip was part of the leg that pushes the pitcher off the ground and the stride hip referred to the leg that made foot contact during the pitch (Figure 1).

**Figure 2:**
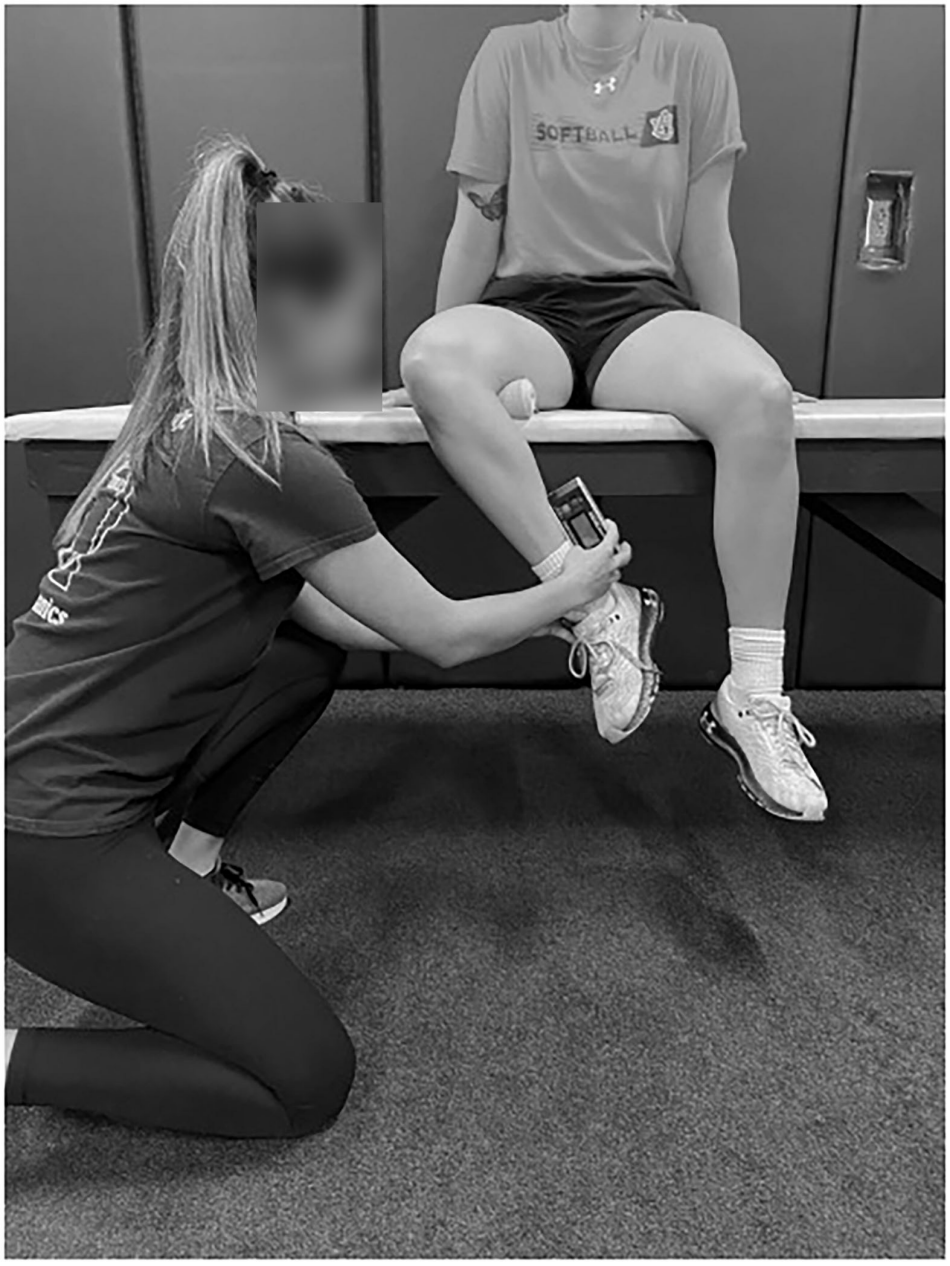
Hip ER ROM measurement.

**Figure 3:**
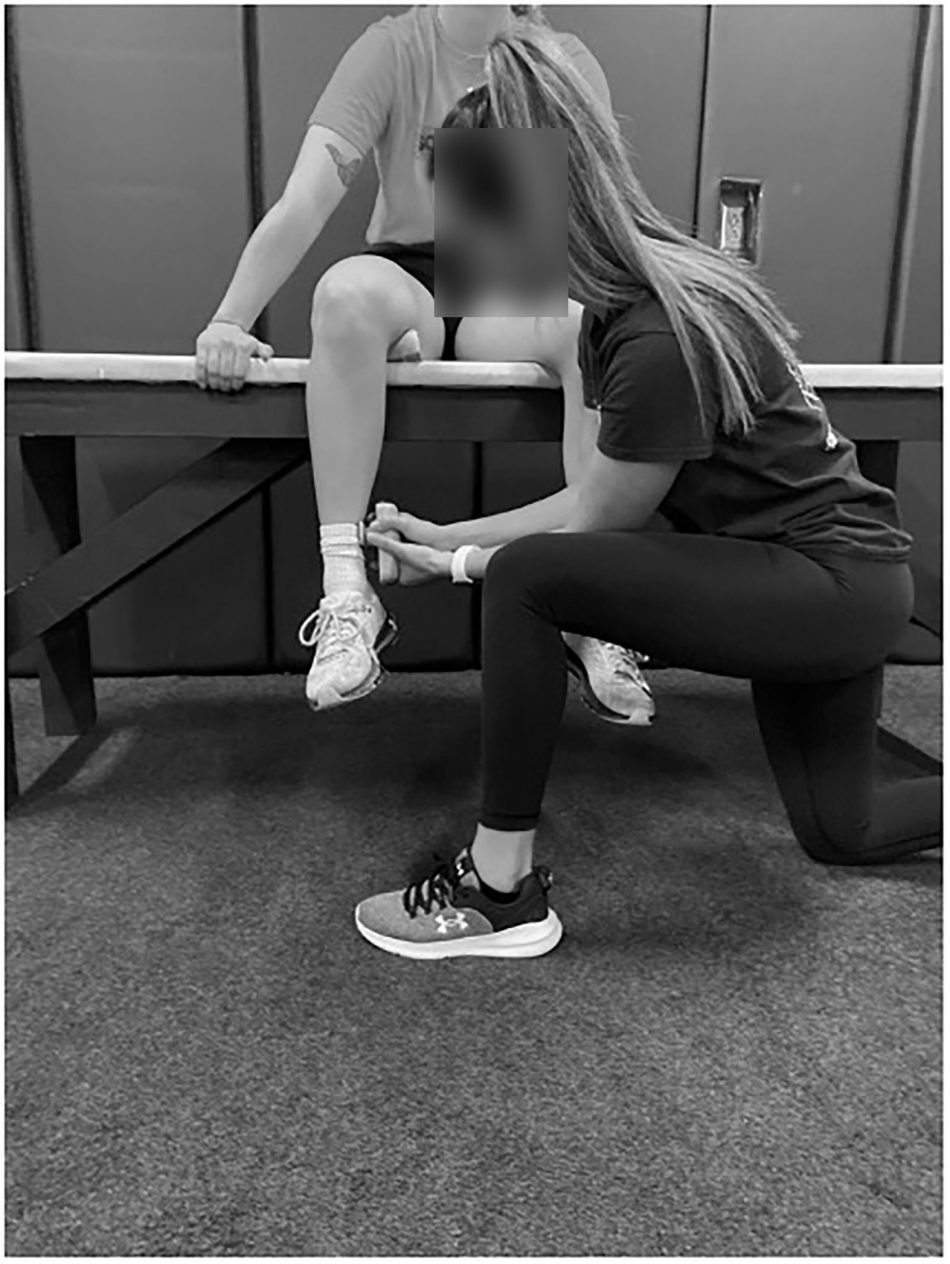
Hip ER ISO measurement.

A handheld dynamometer (Lafayette Instruments, Lafayette, IN, USA) and inclinometer (Fabrication Enterprises, Inc., White Plains, NY, USA) were used to measure ISO and ROM, respectively (Dwelly et al., 2009; Sauers et al., 2014; Oliver et al., 2016, 2019b; Friesen et al., 2019, 2020a). For hip data measurement, pitchers sat on an athletic training table with their hips and knees flexed at 90°. A rolled towel was placed under the distal femur to allow for smooth rotation of the hip joint (see Figure 2). ROM was assessed by having the examiner rotate the shank either toward the contralateral leg (ER measurement) or away from the contralateral leg (IR measurement). The inclinometer was placed on the shaft of the fibula just proximal to the lateral malleolus for IR and on the shaft of the tibia just proximal to the medial malleolus for ER (Friesen et al., 2019). End ROM was determined just prior to when the participants' hip would lift off of the table while seated and with the examiner feeling for firm capsular end feel (Friesen et al., 2019). Hip ISO was also measured in this position (Figure 3). The dynamometer was placed at the same location as the inclinometer and resistance was applied while the participant pushed against the dynamometer and the hip remained in a neutral position during testing.

**Figure 4:**
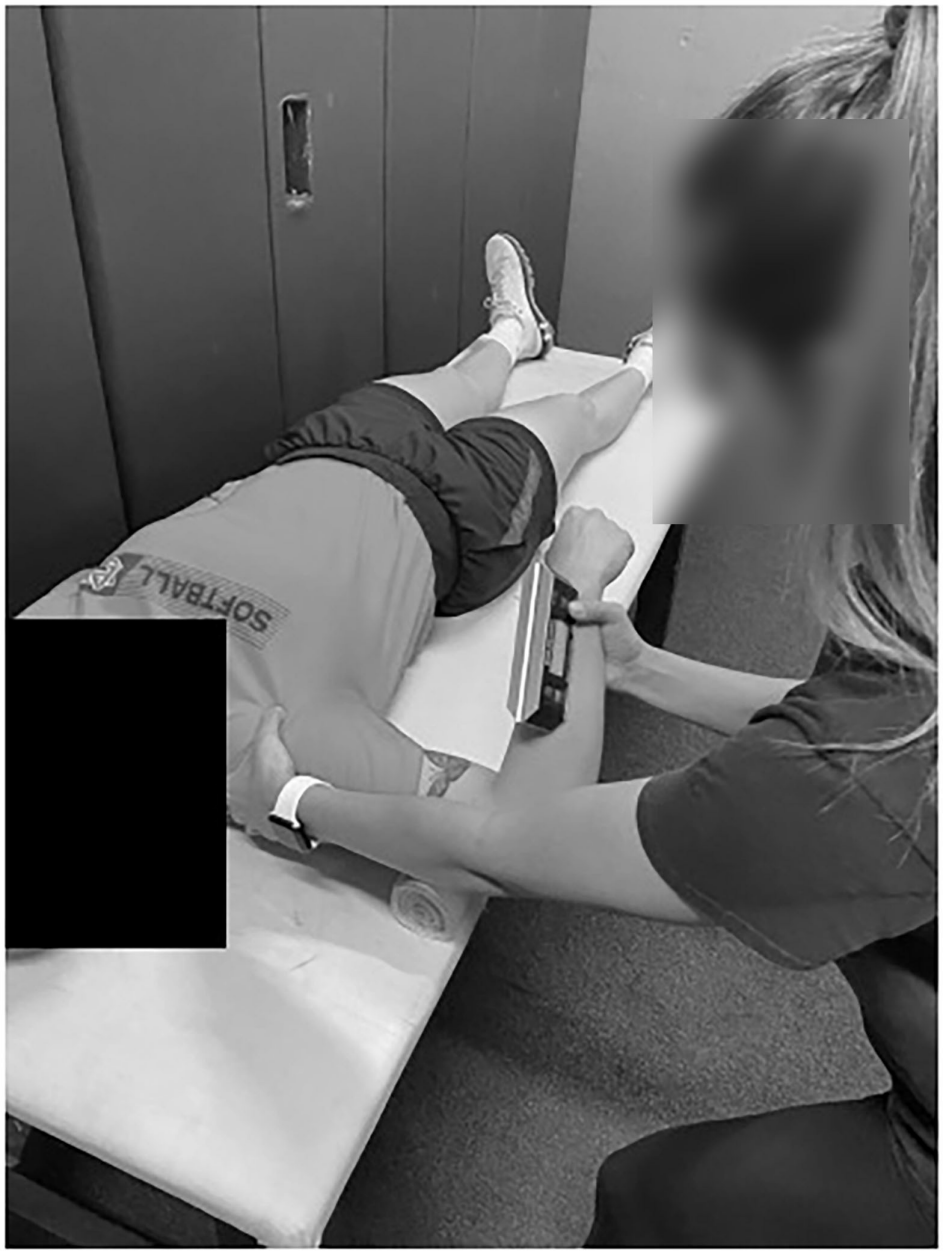
Shoulder IR ROM measurement.

**Figure 5:**
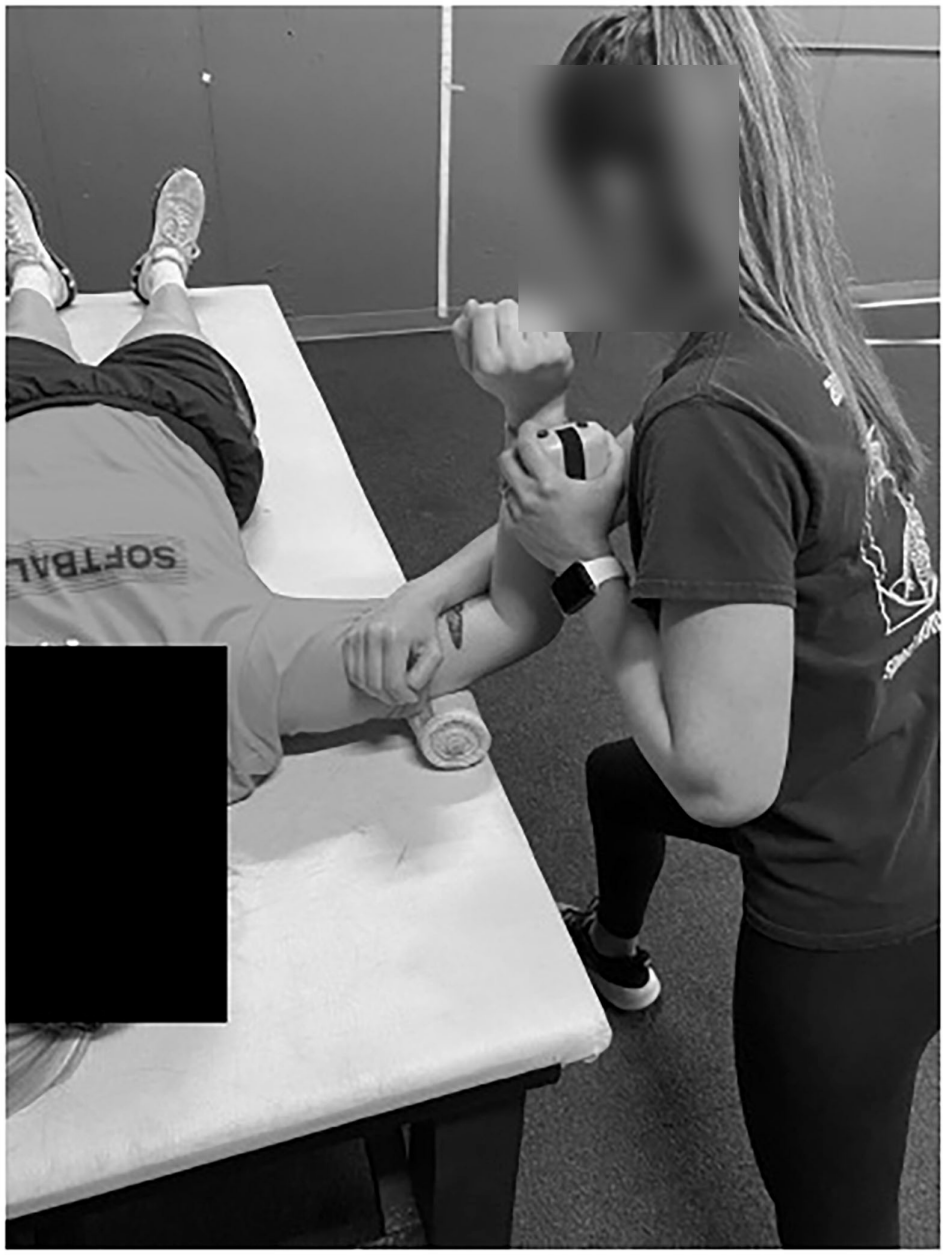
Shoulder ER ISO measurement.

In gathering shoulder measurements, participants lay supine on an athletic training table with their upper arm abducted 90° and their elbow flexed to 90°. Again, a rolled towel was placed under the distal humerus to ensure smooth shoulder movement. The tester used one hand to limit scapular movement while the other hand slowly rotated the forearm either in the direction of the feet (IR measurement) or in the direction of the head (ER measurement). End ROM was determined just prior to when the participants' scapula would lift off the table for IR and at firm capsular end feel for ER (Figure 4). The inclinometer was positioned on the forearm above the styloid process of the ulna for both measurements. ISO was measured in the same way, with the tester maintaining a neutral arm position for the participant, while they applied pressure to the dynamometer (Figure 5). The tester ensured intrarater reliability, with an intraclass correlation coefficient [ICC(3,k)] of 0.92–0.95 for all measurements. Minimal detectable change (MDC) was calculated with a 95% CI to determine clinical significance. Glenohumeral joint IR and ER ROM MDCs were 6.2 and 7.5, respectively. Hip IR and ER ROM MDCs were 6.6 and 4.9, respectively. IR and ER MDCs for ISO measurements were 3.5 and 3.6, respectively, for the glenohumeral joint, and 9.0 and 2.3 for hip IR and ER.

### Statistical Analyses

All statistical analyses were completed in SPSS software package (SPSS Statistics 26 Software, IBM Corp., Armonk, NY, USA). Prior to statistical analyses, all data were checked for normality, linearity, and outliers (defined as those >2 SDs away from the mean). Data were considered normal and linear, with a few outliers present. Analyses were conducted with and without outliers to which no significant differences were observed; therefore, original data with outliers included were used for analyses. Levene's test for equality of variance was conducted and equal variance was consistently observed. Mixed analyses of variance (ANOVA) were conducted to assess the difference between bf% groups and side dominance on several variables, including shoulder and hip external and IR ROM and ISO. Two subsequent mixed ANOVAs were also used to assess arm bone mass and lean mass. Alpha level was set *a priori* to 0.05.

## Results

Means and SDs for each variable are presented in Table 1. The ANOVA assessing IR shoulder ROM revealed a statistically significant interaction between bf% groups and side dominance, F_(1, 39)_ = 14.383, *p* < 0.001, η^2^_*p*_ = 0.269, with an observed power of 0.959. The high bf% group had more dominant shoulder IR ROM than the healthy bf% group (mean difference = 4°) (Table 1). Examination of the healthy bf% group shows that the dominant shoulder displays less IR ROM than the nondominant shoulder (mean difference = 4°), while the high bf% group reveals more IR ROM in their dominant than nondominant shoulder (mean difference = 2°) (Table 1). It is noted that none of these differences are of clinical significance as they are less than the MDC previously calculated.

**Table 1:** Means ± standard deviations for both pitcher groups' hip and shoulder ISO, ROM, and arm lean tissue and bone.

	**Healthy-fat%**		**High-fat%**	
**Variable**	**Dominant**	**Non-dominant**	**Dominant**	**Non-dominant**
Hip IR ROM (°)	30.4 ± 4.0	30.1 ± 3.4	27.3 ± 5.3	28.7 ± 4.6
Shoulder IR ROM^*^	40.7 ± 5.1^a, b^	44.5 ± 4.2^b^	44.8 ± 5.8^a, c^	42.4 ± 6.8^c^
Hip ER ROM (°)	38.0 ± 4.0	38.1 ± 3.8	36.9 ± 4.6	36.3 ± 3.3
Shoulder ER ROM (°)	100.6 ± 14.5	100.7 ± 15.1	94.7 ± 12.2	94.5 ± 17.0
Hip IR ISO^†^	178.2 ± 35.1	170.0 ± 42.8	188.3 ± 42.2	176.1 ± 43.4
Shoulder IR ISO (kgf)	155.7 ± 18.3	155.9 ± 17.8	159.1 ± 24.2	154.9 ± 33.4
Hip ER ISO (kgf)	136.5 ± 26.6	135.3 ± 23.6	138.7 ± 30.4	146.5 ± 33.9
Shoulder ER ISO^†^	168.0 ± 26.0	163.7 ± 23.8	186.1 ± 43.0	171.0 ± 47.0
Arm Lean Tissue (lbs)^†^	5.6 ± 1.0	5.0 ± 1.0	6.3 ± 1.0	5.7 ± 1.1
Arm Bone Tissue (lbs)^†^	0.4 ± 0.1	0.3 ± 0.1	0.4 ± 0.1	0.4 ± 0.1

* *Denotes significant interaction (within a row, same letters denote significant differences)*.

† *Denotes significant main effect for side*.

The ANOVA assessing shoulder ER ISO was significant and revealed a main effect for side, F_(1, 39)_ = 8.133, *p* = 0.007, η^2^_*p*_ = 0.173, with an observed power of 0.794. The dominant/throwing shoulder displayed significantly more ER ISO than the nondominant/glove side (mean difference = 10 kgf). There was also a main effect for side in the ANOVA assessing hip IR ISO, F_(1, 39)_ = 4.635, *p* = 0.038, η^2^_*p*_ = 0.106, with an observed power of 0.556. The dominant/push hip displayed significantly more IR ISO than the nondominant/stride side (mean difference = 10 kgf). No other assessments regarding ROM and ISO were statistically significant.

Examination of bone and lean tissue of the dominant and nondominant arms revealed significant main effects for side dominance. The ANOVA examining bone reported F_(1, 39)_ = 38.620, *p* < 0.001, η^2^_*p*_ = 0.498, with an observed power >0.999. The ANOVA examining lean tissue reported F_(1, 39)_ = 101.869, *p* < 0.001, η^2^_*p*_ = 0.723, with an observed power >0.999. Both bone (mean difference = 0.5 lbs) and lean tissue (mean difference = 0.6 lbs) were heavier in the dominant arm of the pitchers.

## Discussion

The constant asymmetric motions associated with softball pitching and the additional risk for injury associated with body asymmetries (Shanley et al., 2011) warrant investigation to the extent of functional and bodily side-to-side differences among softball pitchers. Furthermore, the added risk of injury for those pitchers who possess more body fat tissue, and the increased injury rates among those who do have excess body fat, emphasizes the need to understand how functional characteristics differ between those with a healthy and high bf%. The main findings of this article show that (1) the side-to-side differences in IR shoulder ROM vary differently between pitcher bf% groups; (2) the dominant side shoulder exhibits greater ER ISO than the nondominant shoulder; (3) the push hip exhibits greater IR ISO than the stride hip; and (4) the dominant side arms display heavier bone and lean tissue. These findings provide insight into the asymmetries of high school softball pitchers and the slight difference in functional characteristics that exist between bf% groups.

### Body Fat Percentage Group Differences

The only functional characteristic to present differences according to bf% group was IR ROM of the shoulder, although it is important to note that these differences were smaller than the MDC calculated previously. Discussion of results should therefore be viewed with this in mind. Data revealed that pitchers with a healthy bf% had less dominant arm IR ROM than the high bf% group (albeit not of clinical significance). The difference between bf% groups suggests that the amount of body fat a pitcher possesses might have a slight influence on functional characteristic adaptations. Loss of IR ROM of the dominant shoulder is common among those athletes who regularly perform throwing tasks (Shanley et al., 2011), but the healthy bf% group displaying less throwing shoulder IR ROM than the high fat% was not expected. Originally, it was hypothesized that the high fat% group might display decreased ROM in general, due to the impingement issues that arise from exhibiting increased fatty tissue. Research points out that those pitchers with a higher body mass index possess less bilateral hip ROM, hypothesized to be the result of greater impinging tissue (Friesen et al., 2020a). It was also expected that the greater mass associated with those in the high bf% group might accrue greater adaptation due to increased loading on the joints. With current data revealing the opposite, it might suggest that the pitchers with a healthy bf% might be accruing greater functional adaptations than the high fat% group. Perhaps healthy fat% pitchers encounter higher repetition and greater pitch counts that might result in greater adaptation (e.g., more throwing shoulder ER ROM and less IR ROM). Conversely, this might suggest that the high fat% group performs less repetitions, which could be either, or both, a cause and effect of carrying extra fat mass. Those who possess extra fat mass might be less active and, as a result, perform fewer and less intense repetitions. Interestingly, a previous report also found that pitchers with higher body mass index did not achieve as much dominant shoulder ER ROM as those with lower body mass index (Friesen et al., 2020a). Therefore, adaptation maybe does not occur the same in a population of pitchers with increased mass and body fat. Future studies are necessary to understand why those softball pitchers with more body fat accrue less throwing shoulder IR ROM as the discussion of causation is merely speculation. It should also be noted that since the acquisition of less IR ROM is a common adaptation of throwers, and our two groups displayed opposite trends of adaptation, future research should account for body mass or bf% as a potential covariate when analyzing functional adaptations of throwers.

### Side-To-Side Differences

Data revealed that there were side-to-side asymmetries in IR ROM between the throwing and glove shoulders. Interestingly, the high bf% group displayed more IR ROM in their throwing shoulder compared to the glove shoulder, opposite of the healthy fat% group. While drastic shoulder asymmetries can lead to injury among throwers (Shanley et al., 2011), the varied asymmetry between bf% groups suggests a potential effect of body fat on shoulder adaptations. However, as asymmetry was present within both populations, we cannot assume that body fat alters symmetry. We can, however, state that body fat might influence functional adaptations, namely, at the shoulder joint. As we know that higher bf% has been demonstrated to be related to higher injury risk (Chalmers et al., 2015; Oliver et al., 2018), this also provides some evidence that perhaps the altered functional characteristics within those with higher body fat could be a potential mechanism for injury; however, more studies are needed to examine mechanisms of injury among those with higher body fat.

Notable side-to-side differences were observed in shoulder ER ISO and hip IR ISO. Based on the pitching motion, asymmetry in IR ISO of the shoulder was expected as another typical adaptation for throwers. During the windmill pitch, the throwing arm shoulder completes rapid IR during the acceleration phase of the pitch (Maffet et al., 1997; Barrentine et al., 1998). To slow the rapid IR, ER strength is necessary to bring the pitcher to a safe stop to finish the pitch. The repeated pitching exposure can strengthen the musculature of the throwing shoulder while in this position, as opposed to the glove arm that performs less overall motion. It was also reported that there was more IR strength in the push hip than in the stride hip. Again, this was expected as the drive off the ground occurs while the pitchers' push hip is internally rotated (Friesen et al., 2020a). In contrast, during the stride, the stride hip needs ER strength more than IR strength to keep the stride leg hip from collapsing and exhibiting greater IR upon aggressive ground contact.

The final findings noted asymmetry in throwing arm lean tissue and bone for both groups of pitchers. As might be expected, bone and lean tissue was heavier within the throwing arm. As this did not vary between pitcher bf% groups, this again highlights the importance of other factors that might influence adaptation to functional characteristics. However, descriptive analysis of the mean and SD data reported in Table 1 shows that the high fat% pitchers did possess greater mean weight of both lean tissue and bone than the healthy fat% group. Perhaps this is an adaptation required to move the generally larger limb of those who are bigger in stature and carry increased body fat.

While research notes that pitchers with increased bf% exhibit higher rates of pitching-related injury (Oliver et al., 2019a; Friesen K. B. et al., 2021) and altered biomechanics during the windmill pitch (Friesen et al., 2020b), there were minimal differences in functional characteristics between bf% groups. There were, however, many asymmetries reported, as could be expected. As bf% does not seem to greatly influence player adaptation, but injury reports suggest an increased injury-susceptibility for larger athletes, additional research is needed to understand the mechanism of injury among softball pitchers. Similarly, more studies are needed to examine the influence of specifically fat mass on softball pitch biomechanics in effort to best inform practitioners working with the development of softball pitchers. Also, this might suggest that other factors such as biomechanics, effort, and practice/game exposure influence functional characteristic adaptations more than the presence of body fat.

### Limitations

This study has some limitations. First, there are potential inconsistencies in maximum effort required during the ISO measurement, and similarly the consistency of the applied rater pressure to the device can vary given the handheld nature of the device. However, all participants were given the same instruction, “to push as hard as possible,” and the rater too gave maximal effort during each measurement. Second, the expertise and development of participants was not controlled; therefore, pitchers with various mechanics and pitching success rates might influence the aggregate data analysis. However, the recruitment sample did come from one general area of the southern United States; therefore, it might be assumed that these players were of a similar skill and competition level. Similarly, the study sample involved a relatively small age range, and it might be expected that older pitchers with more substantial bf% differences could present different and more substantial findings. It is also important to keep in mind that there were no drastic functional characteristic differences between the bf% groups, nor was there a significant number of other variables that differed between bf% groups. Finally, our functional characteristics were measured in static positions and will therefore be limited in their application of the dynamic pitching motion under consideration.

## Data Availability Statement

Data inquiries can be directed to the corresponding author.

## Ethics Statement

The studies involving human participants were reviewed and approved by Auburn University Institutional Review Board. Written informed consent to participate in this study was provided by the participants' legal guardian/next of kin.

## Author Contributions

KF and GO: study conception and design and data collection. KF, AL, KC, and GO: data analysis and interpretation, drafting manuscript, and critical revision. All authors contributed to the article and approved the submitted version.

## Conflict of Interest

The authors declare that the research was conducted in the absence of any commercial or financial relationships that could be construed as a potential conflict of interest.

## Publisher's Note

All claims expressed in this article are solely those of the authors and do not necessarily represent those of their affiliated organizations, or those of the publisher, the editors and the reviewers. Any product that may be evaluated in this article, or claim that may be made by its manufacturer, is not guaranteed or endorsed by the publisher.
